# Melatonin as a Potential Dietary Supplement to Counteract Glyphosate-Induced Decline in Honeybee Populations

**DOI:** 10.3390/insects17020151

**Published:** 2026-01-29

**Authors:** Wenyan Fan, Jingfei Cao, Xinyan Liang, Yiping Wang, Shuhuai Ge, Ting Ji, Jinglan Liu

**Affiliations:** 1 College of Plant Protection, Yangzhou University, Yangzhou 225009, China; 2 College of Animal Science and Technology, Yangzhou University, Yangzhou 225009, China; 3 Jiangsu Province Engineering Research Center of Green Pesticides, Yangzhou University, Yangzhou 225009, China

**Keywords:** honeybees, glyphosate, melatonin, RNA-seq, 16S rRNA

## Abstract

Due to their role as the main pollinators in agricultural environments, honeybees help improve crop quality and yield and also help maintain global and ecosystem biodiversity. However, pesticide exposure during foraging has resulted in declining honeybee populations, and there are increasing concerns about the potential adverse effects of the herbicide glyphosate (Gly) on honeybees. In this study, we show that exogenous melatonin (Mel) alleviated Gly toxicity in honeybees, and we investigated the mechanisms underlying Mel’s effects using RNA-seq and 16S rRNA sequencing. We show that the survival rate of honeybees exposed to Gly increased significantly after pretreatment with 10 mg/L exogenous Mel in the laboratory. RNA-seq showed that genes involved in immunity, digestion, the nervous system, carbohydrate and amino acid metabolism, and development were altered after Gly treatment; interestingly, pretreatment with Mel had a compensatory effect on transcription in response to Gly. 16S rRNA sequencing revealed that Mel treatment dramatically improved the abundance of lactic acid bacteria in honeybees. These findings reveal how melatonin protects honeybee intestines from glyphosate-induced damage, offering practical strategies for honeybee conservation.

## Simple Summary

This study addresses the concerning decline of honeybees, vital pollinators for crops and biodiversity, due to exposure to the herbicide glyphosate. We aimed to test whether adding melatonin could protect honeybees from this toxin. Our results show that melatonin significantly improved the survival of honeybees exposed to glyphosate. It helped restore the normal activity of genes related to health, digestion, and development that were disrupted by the herbicide, and it increased beneficial gut bacteria. We conclude that melatonin alleviates glyphosate-induced harm by shielding the bee’s digestive system and restoring internal balance. These findings offer a practical and promising approach to safeguarding honeybee populations, which is crucial for sustainable agriculture and healthy ecosystems.

## 1. Introduction

The declining honeybee population threatens both global food security and biodiversity. There are many factors contributing to the decline of honeybees, including climate change, global warming, habitat loss, pathogen or parasite infection, and interactions between these factors [1,2,3]. In agroecosystems, reductions in vegetation abundance and diversity have been reported, along with increased pesticide exposure [4]. Long-term exposure to pesticides can have multiple effects on honeybees, including impairments in behavior, physiology, or performance of the colony [5,6,7,8,9]. For example, pesticides can damage honeybee midgut cells, potentially compromising nutrient absorption, survival, and the successful establishment of the colony [2,10,11,12,13]. The guts of social honeybees are colonized by a group of conserved bacterial species, including *Gilliamella apicola*, *Snodgrassella alvi*, *Lactobacillus Firm-4*, *L. Firm-5*, *Bifidobacterium aeroides*, *Bartonella apis*, *Apibacter adventoris*, *Frischella perrara*, and *Parasaccharibacter appium*. Gut microbes help digest food, thereby improving host nutrition; they also help protect the host from pathogens and parasites [14,15]. Honeybees are considered a model organism for microbiome studies because the gut bacteria are major contributors to bee health [16]. Bees exchange bacteria through social behavior, and most honeybees are colonized by the first eight bacterial species listed above, which are considered the core microbiota of the gut [17,18]. The composition of the gut microbiome varies between the larval and adult stages of the honeybee. Microbes are also transmitted from adults to newly emerged honeybees via secretions and feeding activities in the colony [19]. The gut is colonized by a wide variety of microbiota that have diverse roles, including promoting nutrient absorption and digestion, protecting honeybees from pathogens and parasites, improving bee immunity, and ensuring bee health [20,21]. In contrast, under stress, bees rapidly upregulate certain antioxidant and detoxification genes to cope with the challenge, thereby enhancing their oxidative metabolic capacity. HSPs are sensitive biomarkers for stress assessment in various species and are involved in cellular protection and repair during stressful conditions [22]. CYP450s are enzymes involved in xenobiotic detoxification and are produced by many organisms, including insects [23,24]. ZFPs, a class of transcription factors, play important roles in growth, aging, and responses to abiotic and biotic stresses [25].

Glyphosate (Gly) is a systemic herbicide that interferes with the shikimic acid pathway, which is involved in the synthesis of several aromatic amino acids, including phenylalanine, tyrosine, and tryptophan [26]. Studies have found that Gly can impact honeybee foraging behaviors and cognitive abilities [27,28,29]. Although numerous studies have reported the adverse effects of Gly on the growth, physiology, and behavior of honeybees [30,31,32], it is not clear how to reduce the adverse effects of Gly on honeybees.

The hormone melatonin (Mel, N-acetyl-5-methoxytryptamine) is released by the pineal gland and modulates several vital physiological reactions [33], including antioxidant defense systems and mitochondrial-related functions [34]. Oxidative stress can damage lipids and may induce apoptosis [35,36]. Mel provides some protection against oxidative stress by decreasing the accumulation of malondialdehyde (MDA) and reactive oxygen species (ROS) [37,38,39]. Mel has previously been shown to reduce apoptosis induced by paraquat [40], imidacloprid [41], and ochratoxin A [40]. As noted above, the primary mode of action of Gly is to inhibit the synthesis of aromatic amino acids, including tryptophan, a precursor of Mel synthesis [42,43]. Therefore, we hypothesized that Gly could affect Mel synthesis in honeybees, and that adding Mel to the honeybee diet could help reduce Gly toxicity. Although the role of Mel in honeybees is unclear, Mel has been reported to increase the tolerance of *Apis cerana cerana* to low temperatures [44] and to reduce the toxic effects of imidacloprid on *Apis mellifera* [41].

In this study, we compare the responses of *A. cerana cerana* and *A. mellifera* to Gly and Mel. The ecotoxicological effects of Mel on survival, the intestinal microflora, gene expression, and 16S rRNA in honeybees were analyzed at the molecular level. The results provide a molecular basis for assessing the ecological risk of Gly in honeybees. These results provided insights into protective mechanisms of Mel against Gly-induced toxicity in honeybee intestines, and these findings have applied implications for the conservation of honeybees.

## 2. Materials and Methods

### 2.1. Honeybees Breeding and Management

*A. mellifera* and *A. cerana cerana* were maintained at the experimental apiary of Bee Research Institute, College of Animal Science, Yangzhou University (Yangzhou, China). According to previous reports, adult honeybees—recognizable, healthy, and hairy—with pollen on their legs were collected from three healthy outdoor colonies at the hive entrance [22,45]. The honeybees in this experiment were primarily collected from June to October. We collected foraging honeybees during the nectar-rich season compared with other seasons. All honeybees were maintained in an incubator (at a constant temperature of 33 °C with a relative humidity of 70% in a 24 h dark environment), and three honeybees were sampled randomly from each group at the appropriate times.

### 2.2. Gly and Mel Treatment

According to the method described earlier [44], the different amounts of Mel (Macklin, Shanghai, China) (4, 3, 2, 1, and 0.1 mg) were dissolved in 1 mL of ethanol, diluted 100 times, and added to the bee diet. The same volume of the blank solvent (1% ethanol) was used as the control treatment. *A. cerana cerana* and *A. mellifera* were placed in an incubator first fed Mel for 4 days, followed by Gly for 4 days, and an optimal supplemental level of Mel was determined based on bee mortality. The experiment was replicated three times for each concentration. The above honeybees were collected three times from different hives simultaneously.

In honeybee larvae, only the 20 mg/L glyphosate group showed significantly decreased microbiota species diversity and richness, while the 0.8 mg/L and 4 mg/L glyphosate groups showed no significant differences [46]. According to the literature, 10 mg/L was selected as the Gly supplemental level in this experiment [30]. A total of 1800 honeybees were randomly divided into 12 groups (150 per group). Every day, the Gly treatment group was fed 10 mL of fresh 50% sucrose solution containing field-realistic doses of Gly, and the control group was fed 10 mL of fresh 50% sucrose solution without Gly. The above feeding methods lasted for 8 days. Notably, the Mel treatment group was first fed 10 mL of fresh 50% sucrose solution containing optimal Mel content daily for 4 days, followed by 10 mL of fresh 50% sucrose solution containing 10 mg/L Gly daily for 4 days. All groups were reared in an incubator at 33 °C, 70% relative humidity, and 24 h of darkness.

On the 8 th day, according to the literature [47], two kinds of honeybees were randomly captured in each group, disinfected and dissected at a sterile table, and the complete intestinal tracts of honeybees were removed with sterile forceps in groups of 10, and samples were stored at −80 °C for 16S rRNA.

In addition, two kinds of honeybees were randomly captured in each group, and the whole intestines were slowly pulled out from the sting site with tweezers. The whole intestine was observed and photographed under an electron microscope. The remaining honeybee groups were frozen in liquid nitrogen and stored at −80 °C for RNA-seq. Our experiment was performed in three parallel groups to avoid errors and was independently repeated three times.

### 2.3. RNA Extraction and Transcriptome Sequencing

Total RNA was isolated using TRIzol Reagent (Accurate Biology, Changsha, China), and the concentration, quality, and integrity were determined using a NanoDrop spectrophotometer (Thermo Scientific, Waltham, MA, USA). Three micrograms of RNA were used as input material for the RNA sample preparations. Following the manufacturer’s instructions, the PrimeScriptTM RT reagent kit (Vazyme, Nanjing, China) was used to synthesize cDNA from RNA. The sequencing library was then sequenced on the NovaSeq 6000 platform (Illumina, San Diego, CA, USA) by Shanghai Personal Biotechnology Cp, Ltd. (Shanghai, China) The results used the 2^−∆∆Ct^ method and Actin as an internal reference gene. SnapGene 6.0.2 software was used to design the qPCR primers.

### 2.4. DNA Extraction, Amplification, and Sequencing

Gut dissection and DNA extractions of individual guts were performed as outlined in Jones [47]. PCR amplification of the bacterial 16S rRNA genes V3–V4 region was performed using the forward primer 338F (5′-ACTCCTACGGGAGGCAGCA-3′) and the reverse primer 806R (5′-GGACTACHVGGGTWTCTAAT-3′). The quantity and quality of extracted DNAs were measured using a NanoDrop NC2000 spectrophotometer (Thermo Fisher Scientific, Waltham, MA, USA) and agarose gel electrophoresis, respectively. After the individual quantification step, amplicons were pooled in equal amounts, and paired-end 2250 bp sequencing was performed on the Illumina MiSeq platform using the MiSeq Reagent Kit v3 at Shanghai Personal Biotechnology Co., Ltd. (Shanghai, China). Evaluate the quality of the original sequence and identify the low-quality forward and reverse read cutoff. QIIME2 performs quality control and generates an amplicon sequence variant (ASV) signature table.

Quality control functions are used for noise cancellation as well as for the detection and removal of chimeras. To accurately assess microbial community diversity, all samples were diluted to the same depth based on the minimum sequence number. Follow-up analysis was conducted based on the above data. 

### 2.5. Statistical Analysis

The experiment had a completely randomized design. Data presented means ± standard error (SE) (*n* = 3). The statistical analyses were performed using SPSS Statistics (Version 28.0.1.1). Different letters represent significant differences (*p* < 0.05) according to Duncan’s multiple-range test or Student’s *t*-test. The effective Mel concentration for the Mel intake study was determined using one-way analysis of variance (ANOVA), with *A. cerana cerana* mortality as the dependent variable and Mel dose as the independent variable. The α-diversity and β-diversity of the gut microbiome were assessed using the ‘vegan’ and ‘β part’ packages in R software (Version 4.3.2).

## 3. Results

### 3.1. Survival Rate of Honeybees Fed with Different Mel Concentrations

**Figure 1:**
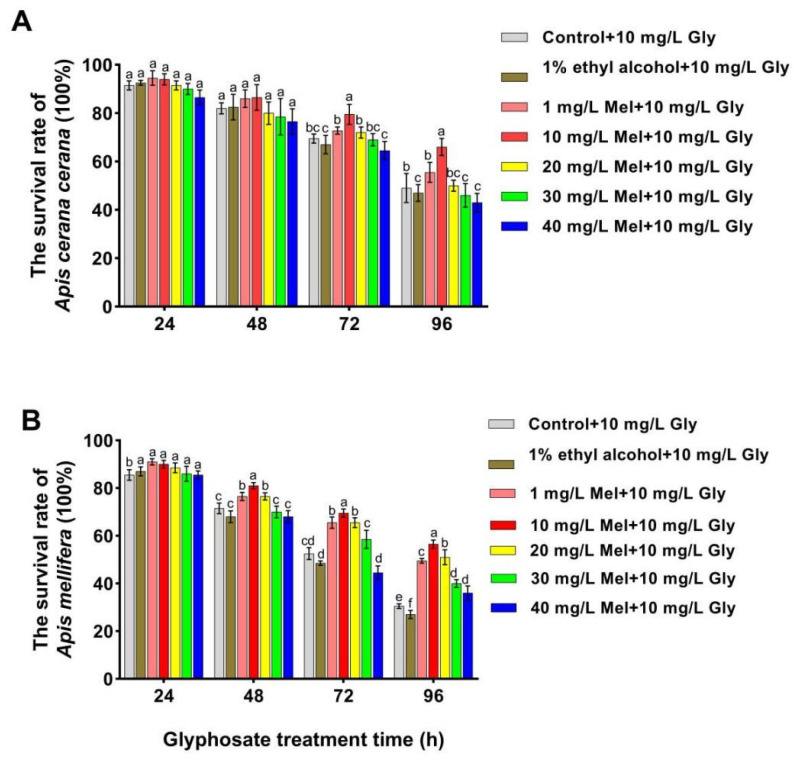
Survival rates of two honeybee species fed with Gly (10 mg/L) and different concentrations of Mel. Panels: Values on the *y*-axis represent the survival rates of (**A**) *A. cerana cerana* and (**B**) *A. mellifera*; values on the *x*-axis represent treatment times. Bars with different colors represent the following treatment groups: gray, 1% ethyl alcohol (ET) + 50% sucrose solution+ 10 mg/L Gly; brown, 1% ET + 50% sucrose solution + 10 mg/L Gly; orange, 1 mg/L Mel + 10 mg/L Gly; red, 10 mg/L Mel + 50% sucrose + 10 mg/L Gly; yellow, 20 mg/L Mel + 50% sucrose + 10 mg/L Gly; green, 30 mg/L Mel + 50% sucrose + 10 mg/L Gly; and blue, 40 mg/L Mel + 50% sucrose + 10 mg/L Gly. In the figure, a, b, c, d, e and f are the grouping markers of statistically significant differences. The value is expressed as the mean ± standard error. One-way analysis of variance, LSD test, *p* < 0.05.

To confirm whether exogenous Mel can mitigate the effects of Gly on honeybees, we analyzed the survival rates of two honeybee species fed with Gly (10 mg/L) and different concentrations of Mel. The survival rates of both *A. cerana cerana* and *A. mellifera* were significantly increased relative to the control at 48–96 h when treated with 10 mg/L Mel (Figure 1). Furthermore, the survival rate of *A. cerana cerana* was higher than that of *A. mellifera*, indicating that Mel had a higher impact on the survival of *A. cerana cerana* than *A. mellifera*. The above results further indicated that exogenous Mel significantly improved honeybee survival, and the survival rate of A. mellifera was significantly lower than that of *A. cerana cerana*. A concentration of 10 mg/L Mel was used in all subsequent experiments.

### 3.2. Microscopy of the Honeybee Gut

**Figure 2:**
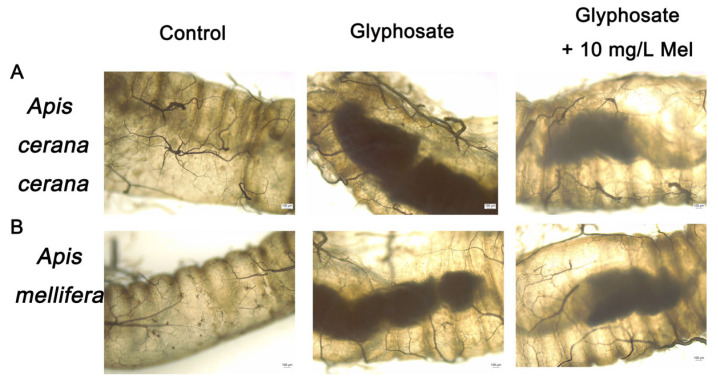
Examination of honeybee intestinal tracts by electron microscopy. Panels: (**A**) *A. cerana cerana*; (**B**) *A. mellifera*. Glyphosate and Mel were added at 10 mg/L, and the control was no Gly.

The intestines’ midgut, ileum, and rectum of the two honeybee species exposed to Gly and exogenous Mel at 10 mg/L were examined by microscopy. In Gly-treated *A. cerana cerana*, the rectum was partially enlarged and darker than that of the control group, whereas it was lighter and less swollen in the Gly + Mel treatment group (Figure 2A). Similarly, rectal enlargement was more severe in the Gly-treated *A. mellifera* group, and the addition of 10 mg/L Mel reduced the rectal size (Figure 2B).

### 3.3. Identification of DEGs in Honeybees Exposed to Gly

The molecular impacts of supplying exogenous Mel to *A. mellifera* and *A. cerana cerana* were investigated by RNA-seq analysis of honeybees fed Mel and/or Gly vs. control bees fed with a sucrose solution. Using threshold values of log_2_ fold-change (FC) > 1.5 and *p* < 0.05, 114 DEGs (Differentially Expressed Genes, DEGs) (53 downregulated and 61 upregulated) were found in *A. cerana cerana* fed Gly as compared to the same species supplied with Mel (Table 1). Seventy-four DEGs (32 downregulated and 42 upregulated) were identified in *A. cerana cerana* fed with Mel as compared to the sucrose control, and 94 DEGs (36 downregulated and 58 upregulated) were observed in *A. cerana cerana* fed with Gly as compared to the sucrose control.

Seventy-eight DEGs (43 downregulated and 35 upregulated) were found in *A. mellifera* fed Gly as compared to the same species supplied with Mel (Table 2). Ninety-eight DEGs (65 downregulated and 33 upregulated) were discovered in *A. mellifera* fed with Mel as compared to those supplied with sucrose, and 101 DEGs (54 downregulated and 47 upregulated) were observed in *A. mellifera* fed with Gly as compared to those fed with the sucrose control.

**Figure 3:**
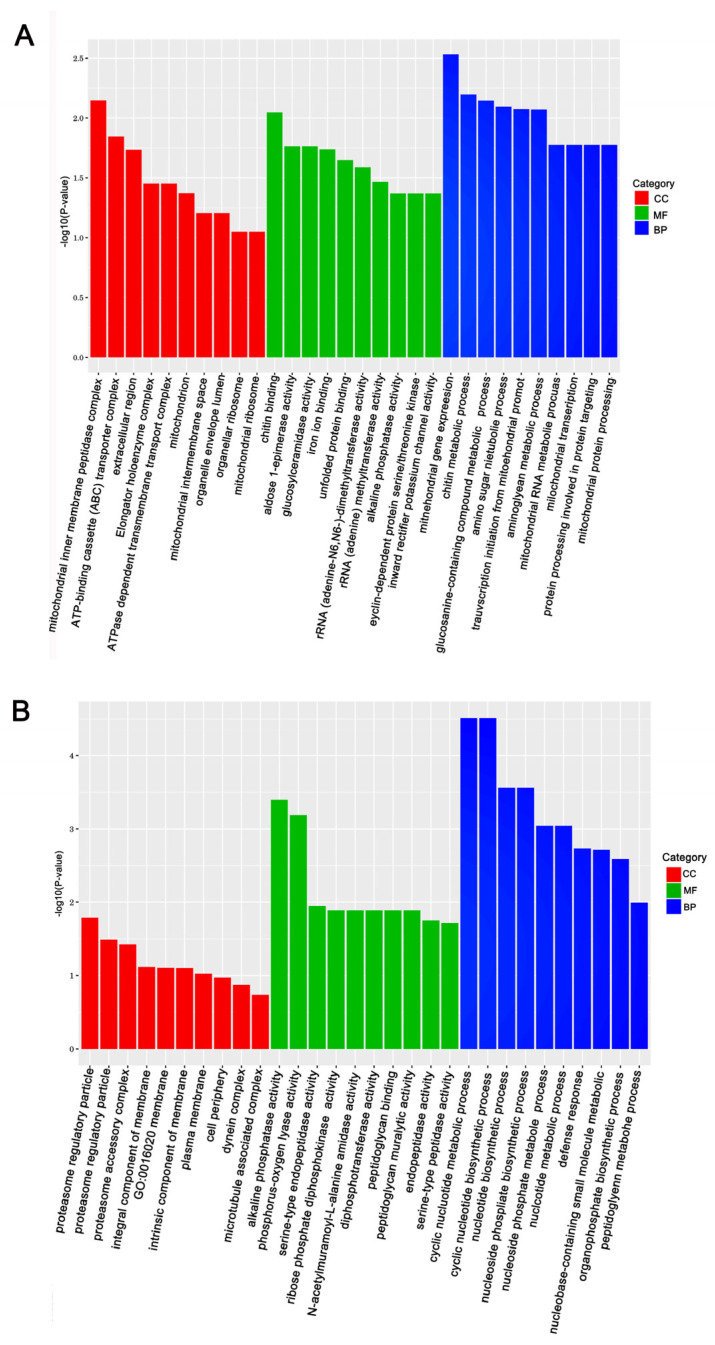
The top 30 enriched GO categories for the DEGs in *A. cerana cerana.* Panels: (**A**) Gly-treated, and (**B**) Mel-treated *A. cerana cerana*. The *y*-axis shows the log_10_ *p*-values, and the *x*-axis indicates the different GO categories.

**Figure 4:**
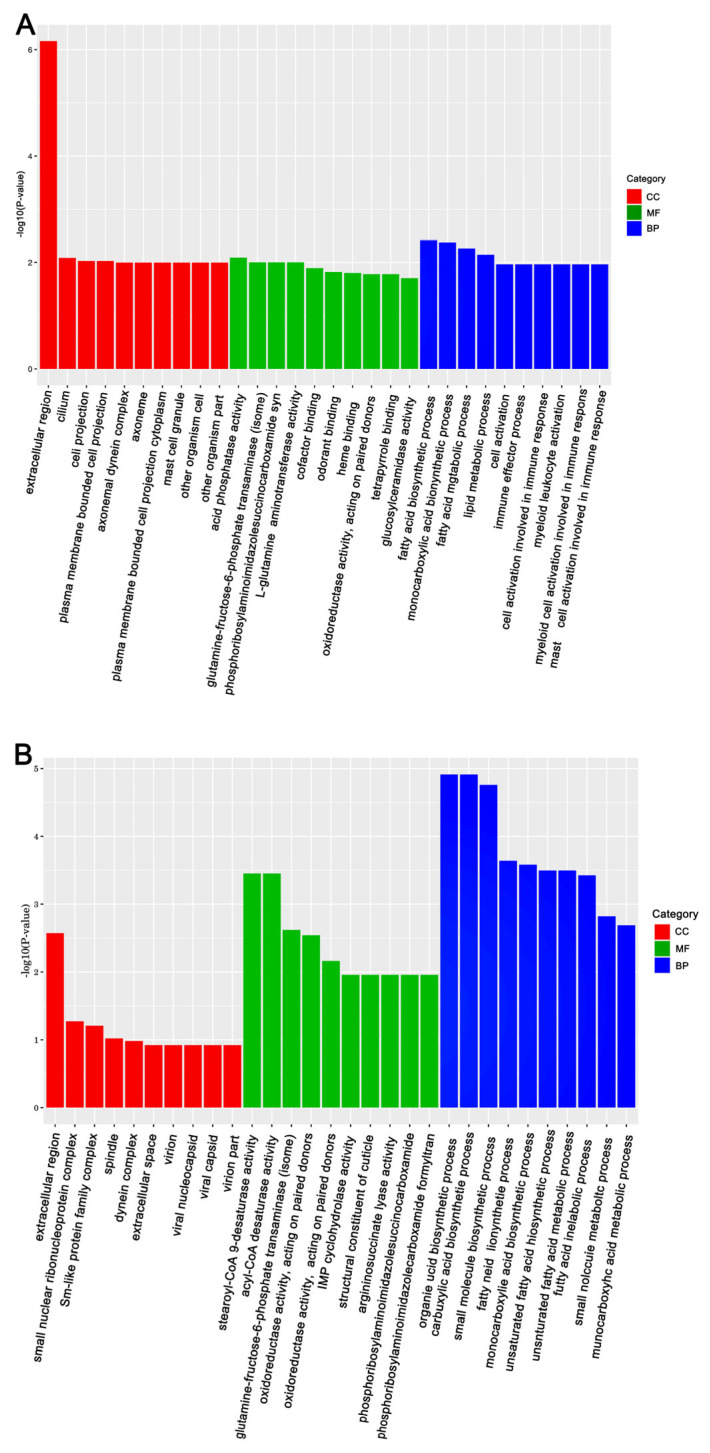
The top 30 enriched GO categories for DEGs in *A. mellifera.* Panels: (**A**) Gly-treated and (**B**) Mel-treated *A. mellifera*. The *y*-axis shows the log_10_ *p*-values, and the *x*-axis indicates the different GO categories.

The KEGG (Kyoto Encyclopedia of Genes and Genomes) and GO (Gene Ontology) databases were used to annotate the DEGs and deduce potential functions. Functional enrichment analysis of DEGs in response to Gly and Mel was conducted with the GO database and compared to the control group. The DEGs were classified using GO into the following groups: cell composition, molecular function, and biological process. Gly primarily affected mitochondrial function and energy metabolism in *A. cerana cerana* and *A. mellifera* (Figure 3A and Figure 4A). In contrast, Mel-treated honeybees showed alterations in nuclear function and nucleotide synthesis (Figure 3B and Figure 4B).

The mapped reads and transcript lengths in the samples were normalized to ensure that the number of fragments accurately reflected transcript levels. The fragments per kilobase of exon per million mapped fragments (FPKM) were used to measure transcript expression levels and to calculate FPKM values for transcripts and genes. False discovery rates (FDRs) and *p*-values were determined to evaluate significance and the reliability of the three treatments in reducing bias. The selected DEGs exhibited FDRs > 0.1 and fold changes > 2 after bias correction.

**Figure 5:**
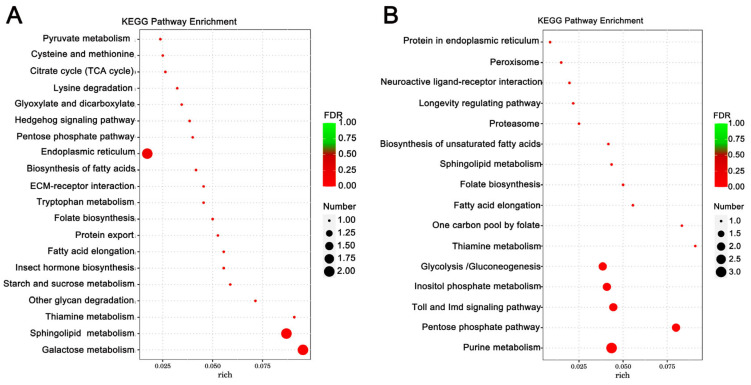
KEGG pathway enrichment scatter plots of *A. cerana cerana* DEGs. Panels: (**A**) Gly-treated and (**B**) Mel-treated *A. cerana cerana*. The lower portion of the scatter plot shows enrichment factors; pathways are shown on the left, and FDR values on the right. Larger enrichment factors indicate greater significance in expression levels. Dot size represents the number of genes, and colors represent different Q values, which are *p*-values.

**Figure 6:**
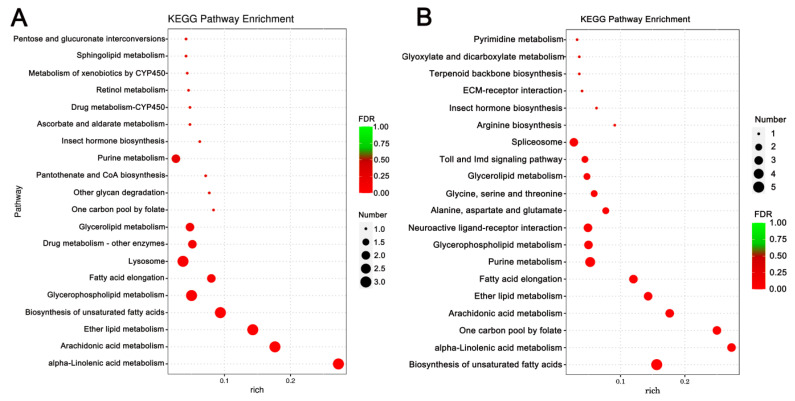
KEGG pathway enrichment scatter plots of *A. mellifera* DEGs. Panels: (**A**) Gly-treated and (**B**) Mel-treated *A. mellifera* DEGs. The lower portion of the scatter plot shows enrichment factors; pathways are shown on the left, and FDR values on the right. Larger enrichment factors indicate greater significance in expression levels. Dot size represents the number of genes, and colors represent different Q values, which are *p*-values.

Enrichment analysis was also conducted using the KEGG database, and the top 10 enriched, significant pathways were chosen for further evaluation. In *A. cerana cerana* treated with Gly, galactose, and sphingolipid metabolism, the differences were highly significant. In Mel-treated *A. cerana cerana*, purine metabolism and the pentose phosphate pathway were the most significant. Arachidonic acid metabolism, α-linolenic acid metabolism, biosynthesis of unsaturated fatty acids, ether lipid metabolism, and glycerophospholipid metabolism were highly represented in *A. mellifera* exposed to Gly (Figure 5). In Mel-treated *A. mellifera*, α-linolenic acid metabolism, biosynthesis of unsaturated fatty acids, and the one-carbon pool by folate were the most altered DEGs (Figure 6).

### 3.4. Expression of Detoxification and Defense-Related Genes

**Figure 7:**
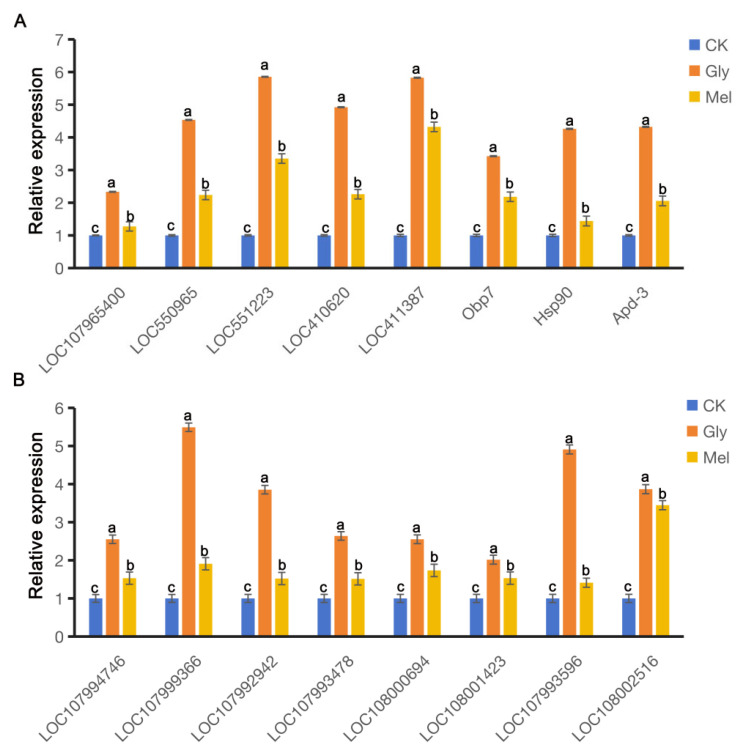
Comparison of gene expression patterns obtained by RT-qPCR. Panels: Values on the *y*-axis represent the relative expression of (**A**) *A. cerana cerana* and (**B**) *A. mellifera*; values on the *x*-axis represent genes. Bars with different colors represent the following treatment groups: blue, 50% sucrose solution; orange, 50% sucrose solution + 10 mg/L Gly; yellow, 10 mg/L Mel + 50% sucrose + 10 mg/L Gly. In the figure, a, b and c are the grouping markers of statistically significant differences. The value is expressed as the mean ± standard error. One-way analysis of variance, LSD test, *p* < 0.05.

RT-qPCR was conducted to verify the RNA-seq results and revealed that many genes were co-regulated in *A. cerana cerana* and *A. mellifera* exposed to Gly and Mel, which was consistent with the RNA-seq results. DEGs involved in detoxification, insecticide stress, and defense were selected from the transcriptome and evaluated by RT-PCR; these genes included cytochrome P450s, ATP-binding cassette (ABC) transporters, heat shock proteins (HSPs), and odorant-binding proteins (OBPs). All this genetic information can be found on NCBI (https://www.ncbi.nlm.nih.gov/) and is described in Table 3. The expression of nine genes was consistently upregulated in both transcriptome sequencing and RT-qPCR (Figure 7).

### 3.5. Profiling the Honeybee Gut Microbiota

**Figure 8:**
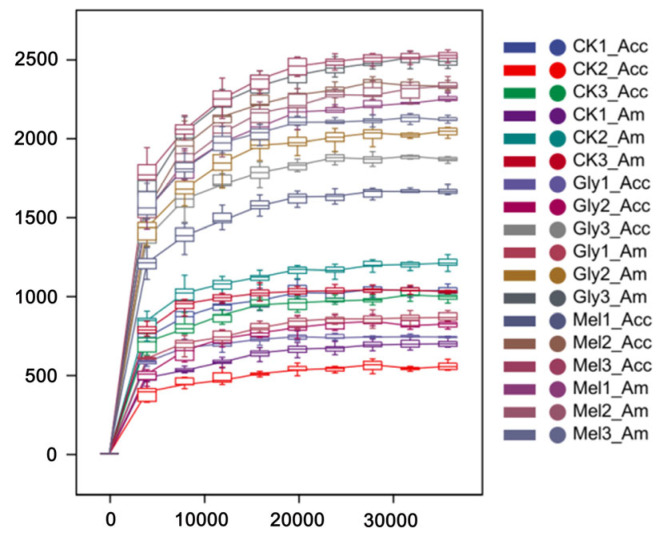
Rarefaction analyses. Rarefaction curves generated from the operational taxonomic units suggested that high sampling coverage was achieved in all samples. Abbreviations: CK, control; Gly, glyphosate; Mel, melatonin; Acc, *Apis cerana cerana*; Am, *Apis mellifera*.

The bacterial communities residing in the guts of *A. cerana cerana* and *A. mellifera* were characterized using the V3 + V4 region of the 16S rRNA amplicon using Illumina sequencing. Paired-end reads were controlled for quality using the Quantitative Insights Into Microbial Ecology (QIIME) pipeline. The analysis included cropping barcodes and primers and filtering low-quality reads and chimeras, which resulted in processed data (Figure 8). The rarefaction curves (Figure 8) for all samples approached the saturation plateau, indicating that the current analysis has sufficient depth to capture most of the microbial diversity.

**Figure 9:**
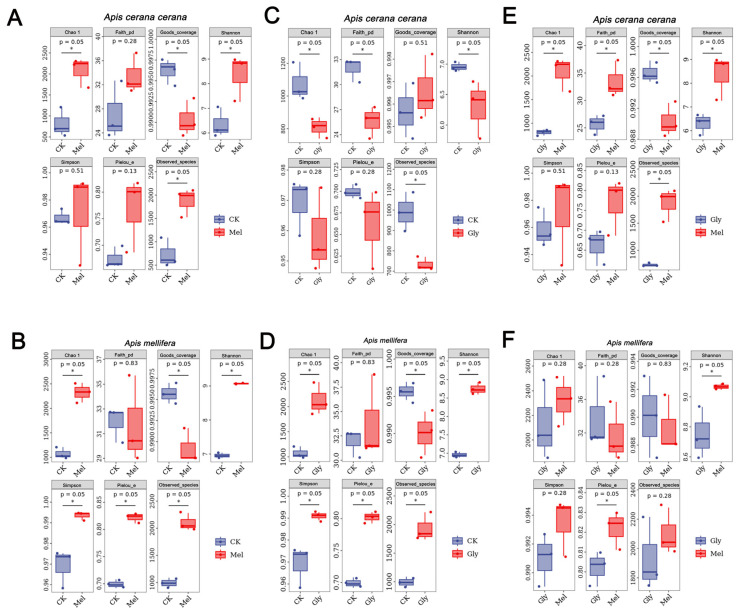
Alpha diversity of honeybee midgut samples. Panels: (**A**), Mel-treated *Acc* vs. CK; (**B**), Mel-treated *Am* vs. CK; (**C**), Gly-treated *Acc* vs. CK; (**D**) Gly-treated *Am* vs. CK; (**E**) Gly-treated *Acc* vs. Mel-treated *Acc*; and (**F**) Gly-treated *Am* vs. Mel-treated *Am*. Abbreviations: CK, control; Gly, glyphosate; Mel, melatonin. * is the grouping markers of statistically significant differences, *p* < 0.05.

ASV-level alpha diversity indices, such as Chao1 richness estimator, Observed species, Shannon diversity index, Simpson index, Faith’s PD, Pielou’s evenness, and Good’s coverage, were calculated using the ASV table in QIIME2, and visualized as box plots. To better understand the characteristics of the midgut microbiota in bees fed with Mel and Gly, the alpha diversity of the microbial community was further assessed using the Kruskal–Wallis and Dunn’s tests. Significant differences were apparent in *A. cerana cerana* treated with Mel as compared with control group (*p* < 0.05) using the Chao1 and Observed species richness indices, as well as the Good’s coverage index, Shannon’s diversity index and Faith’s phylogenetic diversity (PD) index (Figure 9). Furthermore, significant differences (*p* < 0.05) were apparent in *A. cerana cerana* treated with Gly as compared to the control using Chao’s richness index, Shannon’s diversity index, and Faith’s PD index. However, there was a significant difference in the alpha-diversity indices between the Mel and Gly treatments (Figure 9). The deviation from Figure 9 indicated that the coverage of *A. cerana cerana* decreased after Mel treatment. Interestingly, significant differences were observed in both Gly-treated *A. mellifera* and *A. cerana cerana* compared with the control group. For example, the distribution and diversity of *A. mellifera* in the Gly treatment were significantly higher than in the control (Figure 9).

**Figure 10:**
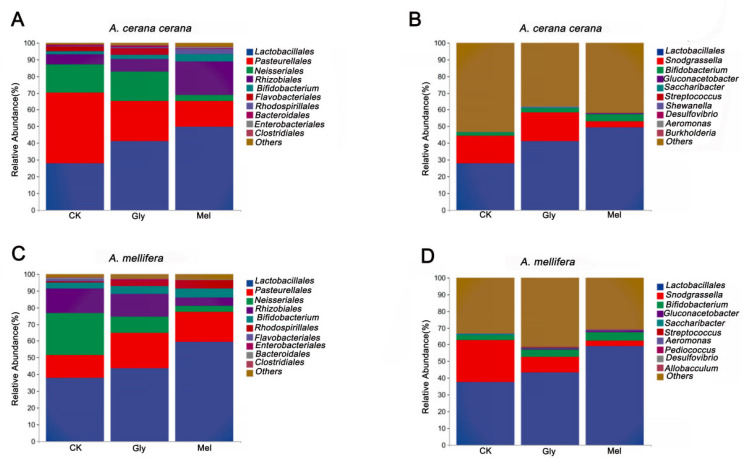
Comparison of major orders and genera. Panels: (**A**,**C**) show the relative abundance of the 10 most abundant bacterial orders in *A. cerana cerana* and *A. mellifera*, respectively. (**B**,**D**) show the relative abundance of the 10 most abundant genera in *A. cerana cerana* and *A. mellifera*, respectively. The *y*-axis represents relative abundance (%), and the *x*-axis shows the different treatments. Abbreviations: CK, control; Gly, glyphosate; Mel, melatonin.

All valid reads were classified (order and genus levels) using QIIME2. The ten most abundant microbial orders in the *A. cerana cerana* gut included Lactobacillales (CK, 27.94%; Gly, 41.21%; Mel, 49.62%), Pasteurellales (CK, 42.15%; Gly, 24.01%; Mel, 15.48%), Neisseriales (CK, 17.08%; Gly, 17.54%; Mel, 3.85%), Rhizobiales (CK, 5.84%; Gly, 7.39%; Mel, 19.97%), Bifidobacteriales (CK, 1.86%; Gly, 2.64%; Mel, 4.31%), Flavobacteriales (CK, 3.13%; Gly, 4.17%; Mel, 4.893 × 10^−5^), Rhodospirillales (CK, 0.18%; Gly, 0.49%; Mel, 3.30%), Bacteroidales (CK, 0.64%; Gly, 0.67%; Mel, 0.15%), Enterobacteriales (CK, 0.03%; Gly, 0.26%; Mel, 0.79%), and Clostridiales (CK, 0.15%; Gly, 0.33%; Mel, 0.23%) (Figure 7A). The ten most abundant microbial orders in the *A. mellifera* gut included Lactobacillales (CK, 37.88%; Gly, 43.76%; Mel, 59.29%), Pasteurellales (CK, 13.53%; Gly, 21.07%; Mel, 18.08%), Neisseriales (CK, 25.36%; Gly, 9.76%; Mel, 3.46%), Rhizobiales (CK, 14.79%; Gly, 13.49%; Mel, 5.32%), Bifidobacteriales (CK, 3.39%; Gly, 4.60%; Mel, 5.28%), Rhodospirillales (CK, 0.76%; Gly, 3.31%; Mel, 4.96%), Flavobacteriales (CK, 1.33%; Gly, 2.539 × 10^−5^; Mel, 1.528 × 10^−5^), Enterobacteriales (CK, 0.22%; Gly, 0.74%; Mel, 0.21%), Bacteroidales (CK, 0.72%; Gly, 0.10%; Mel, 0.13%), and Clostridiales (CK,: 0.38%; Gly, 0.26%; Mel, 0.22%) (Figure 10C).

The ten most abundant bacterial genera in the *A. cerana cerana* gut were *Lactobacillus* (CK, 27.77%; Gly, 41.02%; Mel, 49.24%), *Snodgrassella* (CK, 16.80%; Gly, 17.36%; Mel, 3.74%), *Bifidobacterium* (CK, 1.73%; Gly, 2.54%; Mel, 4.20%), *Gluconacetobacter* (CK, 2.344 × 10^−5^; Gly, 1.475 × 10^−5^; Mel, 0.68%), *Saccharibacter* (CK, 0.16%; Gly, 0.39%; Mel, 0.01%), *Streptococcus* (CK, 0.08%; Gly, 0.09%; Mel, 0.06%), *Shewanella* (CK, 0.04%; Gly, 0.18%; Mel, 0%), *Desulfovibrio* (CK, 0.05%; Gly, 0.12%; Mel, 0.04%), *Aeromonas* (CK, 2.059 × 10^−5^; Gly, 0.15%; Mel, 0.04%), and *Burkholderia* (CK, 0.05%; Gly, 0.05%; Mel, 0.07%) (Figure 7B).

The ten most abundant bacterial genera in the *A. mellifera* gut were *Lactobacillus* (CK, 37.53%; Gly, 43.15%; Mel, 58.99%), *Snodgrassella* (CK, 25.04%; Gly, 9.46%; Mel, 3.35%), *Bifidobacterium* (CK, 3.10%; Gly, 4.39%; Mel, 5.17%), *Gluconacetobacter* (CK, 2.344 × 10^−5^; Gly, 0.66%; Mel, 0.81%), *Saccharibacter* (CK, 0.42%; Gly, 0.36%; Mel, 0.02%), *Streptococcus* (CK, 0.24%; Gly, 0.26%; Mel, 0.10%), *Aeromonas* (CK, 0.15%; Gly, 0.07%; Mel, 0.34%), *Pediococcus* (CK, 0.02%; Gly, 0.10%; Mel, 0.09%), *Desulfovibrio* (CK, 0.07%; Gly, 0.06%; Mel, 0.06%), and *Allobaculum* (CK, 0.02%; Gly, 0.09%; Mel, 0.07%) (Figure 10D).

### 3.6. Differences in the Gut Microbiota of Bees Fed with Sucrose, Gly, and Mel

**Figure 11:**
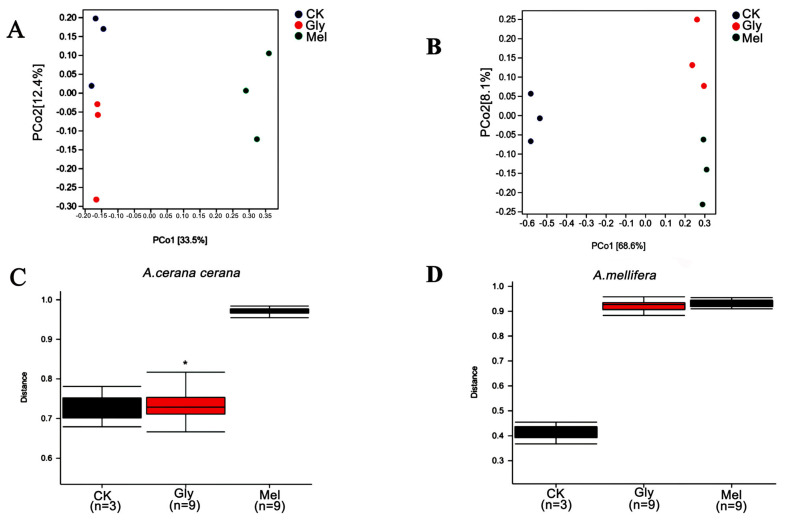
Beta diversity of midgut samples. Principal coordinate analysis (PCoA) of unweighted UniFrac distances for midgut samples obtained from (**A**) *A. cerana cerana* and (**B**) *A. mellifera.* PERMANOVA analysis of microbiota present in (**C**) *A. cerana cerana* and (**D**) *A. mellifera.* * is the grouping markers of statistically significant differences, *p* < 0.05.

To evaluate beta diversity among treatments, principal coordinate analysis (PCoA) was performed using unweighted UniFrac distances among groups. The analysis indicated that the Gly- and Mel-treated bees were different from the sucrose control (Figure 11A,B). Permutational multivariate analysis of variance (PERMANOVA) showed that the microbiota of *A. cerana cerana* was significantly different, but *A. mellifera* was not (Figure 11C,D).

**Figure 12:**
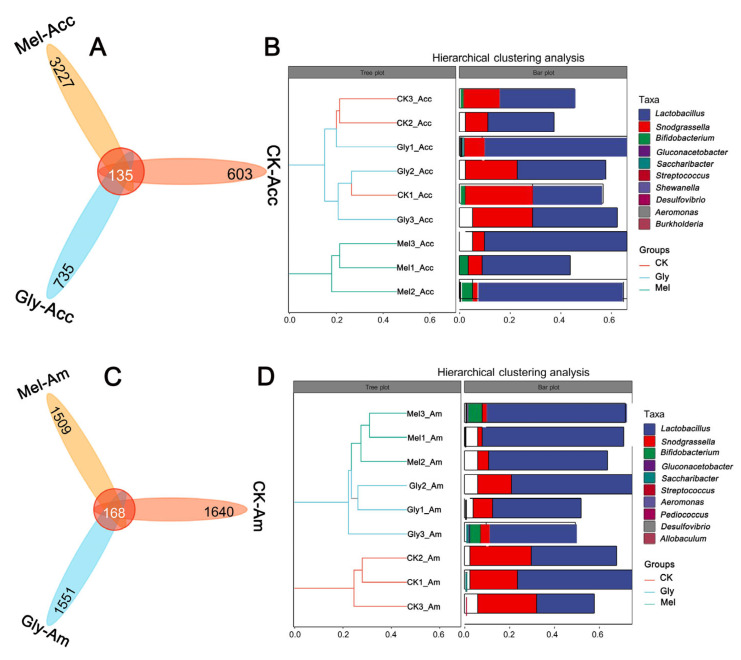
Statistics of OTUs and tags of different samples. The number of midgut OTUs in the three treatment groups is shown in panels (**A**,**B**) for *A. cerana cerana* and *A. mellifera*, respectively. Each color block represents a grouping, and the overlapping area between blocks indicates the common ASV/OTU between the corresponding groups; the number of ASV/OTUs in each block is indicated. Panels (**C**,**D**) show hierarchical clustering trees and stacked histograms of the 10 most abundant microbial genera for *A. cerana cerana* and *A. mellifera*, respectively.

Venn plots were created from cluster analysis of operational taxonomic units (OTUs). Among the Gly, Mel, and control groups of *A. cerana cerana* and *A. mellifera*, 135 and 168 OTUs were common to the guts, respectively (Figure 12A,B). In the *A. cerana cerana* control group, 603 OTUs were unique, fewer than in the Gly- and Mel-treated groups, which had 735 and 3227 OTUs, respectively. In *A. mellifera*, 1640 OTUs were unique to the control group, more than in the Gly- and Mel-treated groups (1551 and 1509 OTUs, respectively).

Hierarchical cluster analysis was used to further analyze the effect of Gly on the gut microbiota of Mel-treated honeybees (Figure 12C,D). Interestingly, we found that the relative abundance of *Lactobacillus* was highest in both the *A. cerana cerana* and *A. mellifera* treated groups. There was little difference in the relative abundance of *Lactobacillus* and *Pasteurellales* in the Gly-treated group.

**Figure 13:**
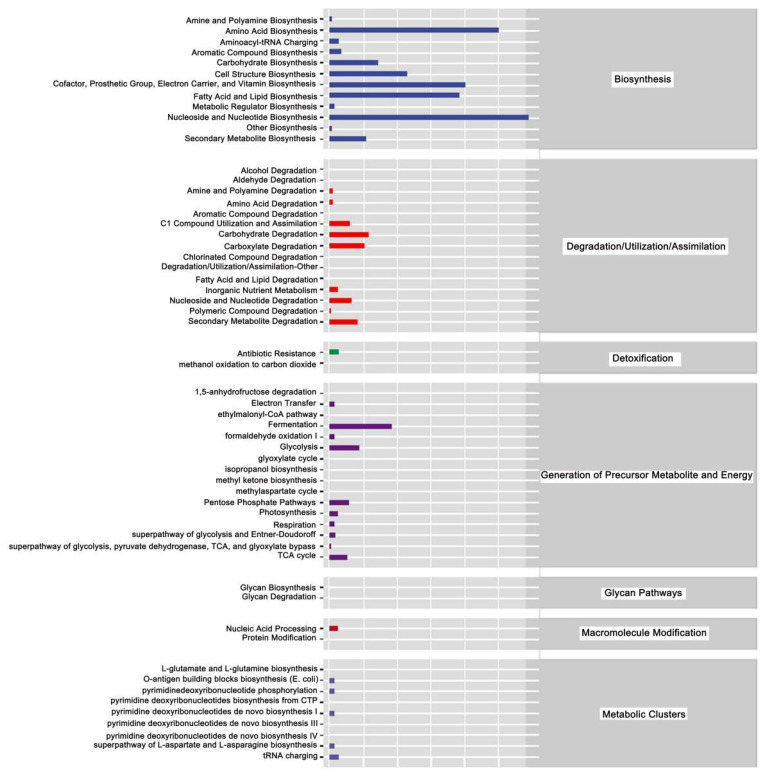
Prediction of metabolic pathways in honeybees treated with Gly and Mel. Metabolic pathways were divided into six categories: biosynthesis; degradation/utilization/assimilation; detoxification; generation of precursor metabolites and energy; glycan pathways; macromolecule modification; and metabolic clusters. Relative abundance is shown at the bottom of the figure, pathways are indicated on the right, and categories are shown on the left.

The above analysis focused on the diversity and species composition of the gut microbiome. For microbial ecology studies, we also paid attention to the functional potential of the flora using Persitional Genescloud. The results indicate microbial functions in biosynthesis, degradation/utilization/assimilation, detoxification, precursor generation for metabolites and energy, glycan pathways, macromolecule modification, and metabolic clusters (Figure 13). The results also suggest microbial functions in the synthesis of amino acids, carbohydrates, cofactors, angle groups, electron carriers, vitamins, nucleosides, and nucleotides. Other functions suggested by this analysis indicate roles for microbial communities in antibiotic resistance and the degradation of aromatic compounds.

## 4. Discussion

Although pesticides are routinely used in agricultural production to control pests and improve crop yields, their impact on pollinators is often overlooked [48,49,50]. Gly is a widely used herbicide [51,52] that indirectly affects pollinators, thereby reducing floral abundance and resource availability [53,54]. Gly affects honeybees not only by impairing the cognitive and sensory abilities of foragers, but also by interfering with gut microbiota, which can reduce survival rate [30,55,56]. Unfortunately, protocols to reduce Gly toxicity to honeybees and efforts to mitigate its effects on honeybees are lacking. The primary aim of this study was to investigate the effects of Mel on survival, digestion, and gut microflora in Gly-treated *A. cerana cerana* and *A. mellifera*.

In this study, KEGG analysis of the two honeybee species showed that Mel impacted energy synthesis (lipid and amino acid metabolism, glycolysis/gluconeogenesis, and the citrate cycle) and detoxification (oxidative phosphorylation and SNARE interactions in vesicular transport). Therefore, we hypothesized that Mel can reduce the effects of Gly by regulating detoxification pathways in honeybees; furthermore, Mel can be catabolized to provide additional energy needed to reduce the effects of Gly. Mitochondria and lysosomes are the main organelles of energy metabolism; the former supply energy, and the latter degrade macromolecules such as proteins, nucleic acids, and polysaccharides. Mitochondria, as the main site of ATP generation, are vital organelles that promote energy conversion and participate in apoptosis [57,58]. Changes in the cellular structure of bee hypopharyngeal glands impair energy metabolism, ultimately affecting the long-term survival of the population [23]. Studies have reported that Gly can downregulate a variety of metabolites related to nutrient metabolism in honeybees, thereby causing malnutrition and affecting the growth and development of honeybees [22]. Further study is needed to determine which metabolic pathways interact synergistically with Mel to regulate the herbicide stress response in bees. It will also be valuable to investigate whether the molecular pathways involved in Mel-mediated defense are conserved in other animals.

Some studies have reported that Mel has a potential detoxification effect on various pesticides, such as deltamethrin [59]; however, the effects of Mel on the physiology of Gly-exposed honeybees have been unclear. In the present study, we show that the addition of Mel to the honeybee diet significantly increased bee survival when compared to the control group (Figure 1). Mel can also increase the expression levels of antioxidant genes in honeybees and their subsequent enzymatic activity [44], which led us to hypothesize that Mel could improve the resistance of honeybees to Gly. In this study, we found that some antioxidant genes, including those encoding HSPs, zinc finger proteins (ZFPs), OBPs, and cytochrome P450 monooxygenases (CYP450s), were upregulated in response to Gly and exhibited antioxidant effects in honeybees, especially after exogenous Mel application. Gly is known to negatively impact associative learning processes and cognitive abilities in honeybees [55]; furthermore, Gly can elicit oxidative stress, which can reduce bee survival. During oxidative stress, cells produce large amounts of ROSs that function in the regulation of apoptosis and cellular survival [60,61]. In order to deal with oxidative stress, the expression of genes encoding antioxidant enzymes increases [62]. Based on our results, we speculate that Mel could increase bee tolerance to Gly by upregulating antioxidant gene expression.

Imbalances in the intestinal microflora of honeybees can result in dysplasia, immune disorders, and various diseases [63]. In this study, the hindgut of honeybees exposed to Gly increased in size and stained deeply, which suggests cellular poisoning after Gly exposure. Interestingly, these conditions were significantly reduced when the two honeybee species were fed Mel and then exposed to Gly, which suggested that Mel significantly improved the gut health of honeybees exposed to Gly. We speculated that the improved gut health was due to changes in the intestinal microbiota, and this hypothesis was tested using 16S rRNA technology. It is well established that host health and gut microbes play an integral role in digestion, nutrient production, immune regulation, and defense against pathogens [63,64]. Gut microbes play a critical role in honeybee growth and health [65], and prior reports have established a reciprocal relationship between the bee diet and gut health. Furthermore, the α-diversity of bee gut microbes was similar to the food consumed by bees, such as pollen and honey [30,66,67]. A significant effect of Gly on honeybee health is its effect on the gut microbiome and the reduced numbers of beneficial bacteria [68]. In this study, the abundance of the *Pasteurellales* (CK, 42.15%; Gly, 24.01%; Mel, 15.48%) was reduced in *A. cerana cerana* as compared with the control group; however, unlike *A. cerana cerana*, the abundance of the *Snodgrassella* (CK, 25.04%; Gly, 9.46%; Mel, 3.35%) was significantly lower in *A. mellifera* as compared to the control.

There are many different bacterial species in probiotic supplements, including those in the genera *Lactobacillus*, *Bifidobacterium*, *Enterococcus*, and *Bacillus*, which are common residents in the honeybee intestine [69,70,71]. There are many studies on *Lactobacillus*, and the presence of this genus is often closely correlated with the intestinal health of the hosts [72,73,74,75,76,77,78,79]. Many research teams have found that feeding honeybees a probiotic mixture of *Lactobacillus* and *Bifidobacterium* can control *Nosema ceranae* infection and increase honey production [75,77]. Although the immune and digestive systems are independent, they have remarkably similar functions in nutrient acquisition and host defense [80]. In this study, the abundance of the *Lactobacillales* was significantly increased relative to the control after bees were exposed to Gly. This difference was more significant when bees were exposed to both Mel and Gly, which is consistent with previous reports. Probiotics are widely used as colony additives to improve honeybee health [69]. *Lactobacillus* can reduce *N. ceranae* spore loads in adult honeybees by upregulating the expression of genes involved in immunity [72,73].

Interestingly, Mel was shown to reshape gut microbes and increase acetic acid levels to regulate lipid metabolism in mice reared on a high-fat diet [81]. The gut microbiota, which can regulate gene expression, plays an important role in host digestion of carbohydrates and proteins [82]. Short-chain fatty acids, important metabolites of gut microbiota, are essential in regulating energy homeostasis [83]. The activities of digestive enzymes (lipase, amylase, and trypsin) directly reflect an organism’s ability to digest and absorb nutrients, which in turn determine rates of growth and development. Interestingly, differences in bacterial colony led to significant differences in amino acid biosynthesis, cofactors, band group, electron carriers, vitamin biosynthesis, fatty acid and lipid biosynthesis, and nucleoside and nucleotide biosynthesis. These effects were significantly enhanced after Mel treatment. In addition, we found that carbohydrate and carboxylate degradation were enhanced, and that glycolysis and the TCA cycle were significantly improved. These results were consistent with the transcriptome sequencing results.

Taken together, our work further reveals that exogenous Mel modulates honeybee exposure to Gly, possibly by increasing total antioxidant capacity by improving the intestinal microbiota, regulating antioxidant genes, and activating energy metabolism and synthesis pathways. Consequently, these results lead us to ask whether melatonin could be applied to other livestock to similarly enhance gut health and promote lactic acid bacteria. This research direction holds significant practical promise. Our results will further enrich the potential regulatory mechanisms of honeybee resistance to pesticides and provide a theoretical basis for the study of Mel in agricultural production.

## Figures and Tables

**Table 1:** Statistical analysis of DEGs in *A. cerana cerana*.

Control	Treatment	Upregulated	Downregulated	Total
Gly_Acc	Mel_Acc	61	53	114
CK_Acc	Gly_Acc	42	32	74
CK_Acc	Mel_Acc	58	36	94

Abbreviations: CK, sucrose control; Gly, glyphosate; Mel, melatonin; Acc, *Apis cerana cerana*.

**Table 2:** Statistical analysis of DEGs in *A. mellifera*.

Control	Treat	Upregulated	Downregulated	Total
Gly_Am	Mel_Am	35	43	78
CK_Am	Gly_Am	33	65	98
CK_Am	Mel_Am	47	54	101

Abbreviations: CK, sucrose control; Gly, glyphosate; Mel, melatonin; Am, *Apis mellifera*.

**Table 3:** Primers used in this study. All genetic information is available on the NCBI.

GeneID	Description	Forward (5′-3′)	Reverse (5′-3′)
107965400	probable cytochrome P450 6a14	CACCATTGGTTTCGGAAATATG	TATCCCAATCATCGGTTCG
550965	probable cytochrome P450 6a14	GGTTAATGATAGAGACTGTTGGCC	CCAGAAACGGAAGAGGCTT
551223	probable cytochrome P450 305a1	CACGTAAATTCGGAGGACAAC	CCATCGTTCATTGTAATTCCTTG
410620	heat shock protein Hsp70Ab-like	CGAGAGAATGGTGAACGAA	CTCATCTTCCATTGTACTCTTC
411387	O-acyltransferase-like protein	GATATCGGTTGGATTCGCTG	CAGAGCTGTCTTTGAGGCACTCT
Obp7	odorant binding protein 7	ACTTTCCGTTGCCGTAAT	GTTCGCCATTTCTACCAA
Hsp90	heat shock protein 90	TGATAGGTCAGTTTGGTGTAGG	TTGGTTCTCCATTGTCAGG
Apd-3	apidermin 3 precursor	TTGACCATCCTCGTAGCGA	CGTTGTTCCTGCGTGACTA
107994746	heat shock transcription factor	GCTTTAGAACAAATATCGCGAG	GTCTATGGTGGATGGTTCATG
107999366	heat shock protein 83	GGAGAGGTTGAAACTTTCG	CAGATGCATTCGAAATCAATTC
107992942	heat shock protein 83	AAGCAGAAGATGTGGAAACTT	TCAAGAGCGTCACTGGAGTTTG
107993478	Chronologically inappropriate morphogenesis	GGCAACCAACAACCATCCC	GTCTCTGCCACCTTCAAGTA
108000694	zinc finger protein 423 homolog	CAGAAGGAAGCAGGCAA	CTCCTCCTCGTCCTGCTT
108001423	probable cytochrome P450 6a14	CTTGGTTAATGATAGAGACTG	TACCCAGAAACGGAAGAGGT
107993596	Golgi-associated RAB2B interactor protein 3-like	GATCGTCCTTGTCGCTG	CTGCTCCATTGTGTCCC
108002516	transcriptional repressor CTCF-like	CAATGAAGATGCTGGAGAGG	CTGTCGCTGCTGAAAGTCT

## Data Availability

The original contributions presented in the study are included in the article, further inquiries can be directed to the corresponding authors.
